# Treatment effects of the differential first-line antiretroviral regimens among HIV/HBV coinfected patients in southwest China: an observational study

**DOI:** 10.1038/s41598-018-37148-8

**Published:** 2019-01-30

**Authors:** Jinhui Zhu, Wenmin Yang, Yuan Feng, Cody Lo, Huanhuan Chen, Qiuying Zhu, Zhiyong Shen, Guanghua Lan, Yi Chen, Zhenzhu Tang, Hui Xing, Yiming Shao, Yuhua Ruan, Liming Li

**Affiliations:** 1 School of Public Health, Peking University, Beijing, China; 2 Guangxi Center for Disease Control and Prevention, Nanning, China; 3 School of Public Health, Yale University, New Haven, USA; 4 Faculty of Medicine, University of British Columbia, Vancouver, BC Canada; 5 State Key Laboratory of Infectious Disease Prevention and Control (SKLID), Collaborative Innovation Center for Diagnosis and Treatment of Infectious Diseases, Chinese Center for Disease Control and Prevention (China CDC), Beijing, China

## Abstract

HIV with HBV co-infection can result in greater HIV-related immunosuppression, morbidity and mortality. Currently, there are few studies to evaluate direct treatment effects on mortality and attrition rates between first-line antiretroviral therapy (ART) based-on tenofovir (TDF) and/or lamivudine (3TC) in a real-world setting. We used Cox proportional hazard models to evaluate direct treatment effects of the first-line ART containing stavudine (d4T), azidothymidine (AZT) and TDF on death and attrition among HIV patients with HBV coinfection. A total of 3912 patients met study eligibility criteria. The overall mortality rate and attrition rate was 2.85 (95% CI: 2.55–3.16) and 8.87 (95% CI: 8.32–9.41) per 100 person-years, respectively. The ART containing TDF had a significantly lower risk of death [adjusted hazard ratio (AHR) = 0.58, 95% CI: 0.44–0.77] when compared to the ART containing d4T, but the risk of death was not significantly different when compared to the ART containing AZT (AHR = 0.91, 95% CI: 0.69–1.20). Patients with HIV/HBV coinfection receiving the ART containing TDF had significantly lower risk rates of attrition compared to those receiving the ART containing d4T (AHR = 0.72, 95% CI: 0.60–0.86) or AZT (AHR = 0.67, 95% CI: 0.58–0.77). Compared with the ART containing d4T, the ART containing AZT was significant and not significant associated with a lower risk of death and attrition, respectively. The ART containing TDF had significant effects on both of death and attrition among HIV patients with HBV coinfection.

## Introduction

Since 1996, the availability of combination antiretroviral therapy (ART) has dramatically reduced HIV-related mortality and morbidity and improved the quality of life for patients^1–4^. Most developing countries have now initiated ART treatment programs, with countries such as South Africa and Brazil being the earliest adopters, many of these programs were scaled-up after the 2003 recommendation of the Joint United Nations Programme on HIV/AIDS (UNAIDS) and World Health Organization (WHO) “Three by Five” initiative^5^. One such program was China’s National Free Antiretroviral Treatment Program (NFATP) which began as a pilot in the early 2000s, and was scaled-up in 2003^6,7^. By the end of 2016, a total of 489,411 HIV patients across the China were receiving free antiretroviral treatment^8^. Many observational cohort studies have found that the NFATP has successfully increased life expectancy, decreased AIDS-related morbidity and mortality, and had significant effects on viral suppression, drug resistance and treatment for HIV prevention among Chinese HIV patients^6,7,9–17^. As of 2017, there are an estimated 21 million HIV patients receiving antiretroviral therapy worldwide, enabling them the opportunity to live full and productive lives^18^.

HIV and hepatitis B virus (HBV) infection is estimated at 35 million and 400 million worldwide respectively^19,20^. Due to HIV and HBV sharing similar pathways of transmission, many people living with HIV are co-infected with HBV, many of whom reside in Asian countries such as China^21,22^. HIV and HBV co-infection can greatly increase HIV-related immunosuppression, morbidity and mortality relative to either infection alone. The nucleoside reverse transcriptase inhibitors such as 3TC and TDF are effective for treatment of both HIV and HBV^20^. The World Health Organization (WHO) recommends initiation of ART for HIV/HBV co-infected patients and the NFATP recommended to switch the first-line regimen from didanosine to 3TC in 2008^23,24^. After that, the first-line regimens were AZT/d4T + 3TC + NVP in China. Patients with HBV treated only with 3TC quickly develop resistance so TDF is typically given in combination^25,26^. In 2012, TDF/AZT + 3TC + EFV/NVP regimens were introduced as the first-line treatments of the NFATP^24^. Patients with HIV and HBV coinfection treated with antiretroviral therapy containing TDF/3TC demonstrated the safety and effectiveness of the regimen^6,27–29^. However, there are currently few studies to investigate associations between ART based-on TDF and/or 3TC with direct treatment effects. We used the NFATP database from China to evaluate treatment effects of the ART containing D4T (d4T + 3TC + NVP), AZT (AZT + 3TC + EFV/NVP) and TDF (TDF + 3TC + EFV/NVP) on death and attrition among patients co-infected with HIV/HBV in an observational cohort study.

## Results

### Baseline characteristics of study patients

A total of 41,071 patients with HIV initiated combination antiretroviral therapy between 2010 and 2014 in Guangxi, China. Many patients (37,159) were excluded due to no HBV infection (25,639), no HBV testing results (11,056), lack of follow-up data (5 patients had no follow up besides baseline visit), being less than 18 years old (82), and not initiating a standard first-line treatment regimen (377). A total of 3,912 patients met study eligibility criteria and entered this observational cohort study analysis among patients with HIV/HBV coinfection.

Table 1 presents baseline characteristics of the study patients. The proportion of study patients whose baseline age ≥50 years was 24.6%. 73.3% were male and 67.2% of study patients were married. The majority (84.3%) of study patients were infected through heterosexual intercourse, followed by homosexual intercourse (1.8%), intravenous drug use (11.8%) and unknown/other (2.2%). The prevalence of HCV infection was 13.5%. The proportion of study patients with CD4 cell count before ART ≤ 350 cells/μL were 88.3% and 44.9% of study patients were with WHO clinic stage III/IV before ART. The initial regimens used by study patients were: the ART containing D4T (d4T + 3TC + NVP) (14.8%), the ART containing AZT (AZT + 3TC + EFV/NVP) (29.2%) and the ART containing TDF (TDF + 3TC + EFV/NVP) (56.0%). The proportion patients who were currently using a first-line ART regimen was 90.2%.

**Table 1:** Baseline characteristics of HIV patients with HBV coinfection who starting ART between 2010 and 2014 in Guangxi, China.

Variable	Number	%
Total	3912	100.0
Age (years)		
18–50	2948	75.4
≥50	964	24.6
Sex		
Male	2869	73.3
Female	1043	26.7
Marital status		
Married	2628	67.2
Other	1284	32.8
Route of HIV transmission		
Heterosexual intercourse	3297	84.3
Homosexual intercourse	69	1.8
Intravenous drug use	461	11.8
Other	85	2.2
HCV infection		
No	3040	77.7
Yes	530	13.5
Missing	342	8.7
CD4 cell count (cells/mm^3^) before ART		
≤350	3454	88.3
>350	408	10.4
Missing	50	1.3
WHO clinic stage before ART		
I/II	2157	55.1
III/IV	1755	44.9
Initial first-line ART regimen		
ART containing D4T	578	14.8
ART containing AZT	1142	29.2
ART containing TDF	2192	56.0
Current ART regimen		
First-line ART	3530	90.2
Second-line ART	382	9.8
Year of ART initiation		
2010	620	15.8
2011	761	19.5
2012	771	19.7
2013	832	21.3
2014	928	23.7

### Mortality rates and effects of initial ART regimen on death in study patients

Among all study patients, 315 deaths occurred, 253 patients stopped ART, and 726 patients were lost to follow-up. A total of 11042.40 person-years were followed. The overall mortality rate was 2.85 (95% confidence interval [CI]: 2.55–3.16) per 100 person-years among all study patients. The mortality rate was 4.42(95% CI: 3.50–5.34), 2.44 (95% CI: 1.95–2.93) and 2.58 (95% CI: 2.16–2.99) per 100 person-years among all study patients initiating ART containing D4T, AZT and TDF, respectively (Table 2).

**Table 2:** Mortality rates in HIV patients with HBV coinfection starting ART between 2010 and 2014 in Guangxi, China, by initial ART regimen.

Variable	Number	Deaths	Person years	Deaths per 100 person years (95% CI)
Total	3912	315	11042.40	2.85 (2.55–3.16)
Initial first-line ART regimen				
ART containing D4T	578	85	1922.51	4.42 (3.50–5.34)
ART containing AZT	1142	90	3689.36	2.44 (1.95–2.93)
ART containing TDF	2192	140	5430.54	2.58 (2.16–2.99)

Table 3 presents adjusted effects of initial ART regimen on death in study patients. Comparing the ART containing D4T in the adjusted models, the ART containing AZT and TDF was significantly (AHR = 0.65, 95% CI: 0.48–0.89 and AHR = 0.58, 95% CI: 0.44–0.77) associated with a lower risk of death, respectively. In the adjusted model for sub-group analysis of effects of initial ART regimen on death, there was no significant differential between the ART containing AZT and the ART containing TDF.

**Table 3:** Effects of initial ART regimen on death in HIV patients with HBV coinfection starting ART between 2010 and 2014 in Guangxi, China.

Variable	AHR^*^ (95% CI)	P-value	AHR^*^ (95% CI)	P-value
Initial first-line ART regimen				
ART containing D4T	Reference			
ART containing AZT	0.65 (0.48–0.89)	0.006	Reference	
ART containing TDF	0.58 (0.44–0.77)	<0.001	0.91 (0.69–1.20)	0.502

^*^AHR = adjusted hazard ratio; covariates of the adjusted model were included: demographic variable (age, sex, marital status) and clinic variable (route of HIV infection, CD4 count before ART, WHO clinic stage before ART, year initiated ART).

### Attrition rates and effects of initial ART regimen on attrition in study patients

Among all study patients, 979 attritions occurred, including 253 patients stopped ART and 726 patients were lost to follow-up. The overall attrition rate was 8.87 (95% CI: 8.32–9.41) per 100 person-years among all study patients. The attrition rate among all study patients initiating the ART containing D4T, AZT, and TDF was 9.31(95% CI: 7.98–10.64), 10.14(95% CI: 9.14–11.14) and 7.84 (95% CI: 7.12–8.57) per 100 person-years respectively (Table 4).

**Table 4:** Attrition rates in HIV patients with HBV coinfection starting ART between 2010 and 2014 in Guangxi, China, by initial ART regimen.

Variable	Number	Attrition	Person years	Attritions per 100 person years (95% CI)
Total	3912	979	11042.40	8.87 (8.32–9.41)
Initial first-line ART regimen				
ART containing D4T	578	179	1922.51	9.31 (7.98–10.64)
ART containing AZT	1142	374	3689.36	10.14 (9.14–11.14)
ART containing TDF	2192	426	5430.54	7.84 (7.12–8.57)

Table 5 presents adjusted effects of initial ART regimen on attrition in study patients. Compared to the ART containing D4T in the adjusted models, the ART containing AZT and TDF was not significantly (AHR = 1.10, 95% CI: 0.92–1.31) and significantly (AHR = 0.72, 95% CI: 0.60–0.86) associated with higher and lower risk of attrition, respectively. Compared with the ART containing AZT in the adjusted model for sub-group analysis, the ART containing TDF (AHR = 0.67, 95% CI: 0.58–0.77) was significantly associated with lower attrition.

**Table 5:** Effects of initial ART regimen on attrition in HIV patients with HBV coinfection starting ART between 2010 and 2014 in Guangxi, China.

Variable	AHR^*^ (95% CI)	P-value	AHR^*^ (95% CI)	P-value
Initial first-line ART regimen				
ART containing D4T	Reference			
ART containing AZT	1.10 (0.92–1.31)	0.311	Reference	
ART containing TDF	0.72 (0.60–0.86)	<0.001	0.67 (0.58–0.77)	<0.001

^*^AHR = adjusted hazard ratio; covariates of the adjusted model were included: demographic variable (age, sex, marital status) and clinic variable (route of HIV infection, CD4 count before ART, WHO clinic stage before ART, year initiated ART).

## Discussion

In this observational cohort study, we found the overall mortality rate was 2.85 per 100 person-years among patients with HIV/HBV coinfection starting ART between 2010 and 2014 in Guangxi, China. The low mortality rate in our study was comparable to treatment effects seen in other developing and developed countries^7,21,26–30^. This demonstrated a rapid but successful scale up of China’s NFATP. Similarly, low mortality rates were also observed among HIV patients with HBV coinfection starting the ART containing D4T (d4T + 3TC + NVP) and AZT (AZT + 3TC + EFV/NVP) in our study. In China, initial ART regimens include 3TC as it is effective against both HIV and HBV. Our study findings also support reports that suggest the use of 3TC as a backbone of cART (combination ART) when treating patients with HIV who also test positive for HBsAg^31^.

Our large observational cohort study found that the ART containing TDF (TDF + 3TC + EFV/NVP) was significant associated with lower mortality risk among patients with HIV/HBV coinfection. The comparison with AZT-containing regimens shows a reduction in the mortality risk although it is not statistically significant. Previous studies have not reported such a direct effect of TDF-based regimens on mortality amongst patients with HBV/HIV co-infection^7,21,26–32^. TDF entered the antiretroviral market in 2002, which is a safe, efficacious, and cost-effective alternative to AZT and d4T-based ART regimens^33^. TDF was active against HBV and an important component of HBV suppressive ART which alone seems to lower HBV DNA as effectively as 3TC + TDF^26^. Currently, the WHO recommends HBV/HIV co-infected patients start an ART regimen containing TDF and China’s NFATP uses TDF + 3TC + EFV/NVP as the current initial first-line regimens^23,24^. Compared with the ART containing D4T in our study, the ART containing AZT was significant associated with lower death. This study results support China’s NFATP to remove D4T due to side effects and efficacious^24^. Prior to this study, there was no observational data to support these recommendations in a real-world cohort.

Our study showed the overall attrition rate was 8.87 per 100 person-years among all study patients, which was higher than that in high-income settings, but lower than that in other low-income settings^34,35^. Compared with the ART containing D4T (d4T + 3TC + NVP) and AZT (AZT + 3TC + EFV/NVP), the ART containing TDF (TDF + 3TC + EFV/NVP) was associated with lower attrition risk among patients with HIV/HBV coinfection in this study. TDF has replaced both D4T and AZT in China, due to its more favorable toxicity profile, dosing, and cost-effectiveness^24^. The D4T regiment has salient side effects which can affect treatment outcomes. These findings also provide additional evidence to strongly support China’s NFATP initiating TDF + 3TC + EFV/NVP among all HBV/HIV co-infected individuals since 2012, as advised by WHO guidelines^23,24^. Considering moderate attrition, it is essential that adherence is promoted amongst patients with HIV/HBV and physicians providing HIV care and ART receive comprehensive education and training to further improve clinical outcomes.

Our results should be interpreted within the limitations of this observational cohort study. First, selection bias of study participants in this cohort study may have been an issue, although we used the adjusted models to control for potential confounders. Due to our study did not include HIV diagnosed individuals but did not initiate ART, it is possible that individuals with poorer health-seeking behaviors were underrepresented in the study. Second, HBV infection was tested using an HBsAg ELISA which may not represent active HBV status. This definition could lead to overestimation of HBV infection, which may reduce statistical significance. Third, patients who were lost to follow-up might have different treatment outcomes compared to patients who remained in the study, such as high mortality and viral load rebound due to stop ART treatment. Fourth, due to this cohort study being conducted in Guangxi, study results might not be fully representative of China or other countries providing ART.

In conclusion, this is the first large-scale observational cohort study in a low- or middle-income countries to assess the effects of initial ART regimen on death and attrition in HIV patients with HBV coinfection. China aims to achieve the UNAIDS “90-90-90” target for significant reductions in HIV-related mortality and HIV new infection^36^. Based on comprehensively considering different mortality and attrition rate among HIV patients received the ART containing D4T, AZT and TDF (TDF + 3TC + EFV/NVP). We believe these findings demonstrating the utility of ART regimens containing TDF in a prospective observational cohort in China and will help optimize first-line antiretroviral therapy among patients with HIV/HBV coinfection in China and other countries.

## Materials and Methods

### Study design and study participants

This observational cohort study was conducted in the Guangxi Zhuang Autonomous Region of southwest China. In 2016, Guangxi represented 10% of the total number of nationally reported HIV cases and this region has accumulated the third highest number of HIV cases reported in China. Heterosexual transmission was the main mode of transmission accounting for 93% of reported HIV cases in Guangxi^37^.

Patients with HIV/HBV co-infection from the NFATP database were collected from 2010 to 2016, the characteristics of this database have been previously described^6,7,10,24^. Physicians administering the ART at the local hospitals manage case report forms at the time of initiating ART and follow-up at 0.5, 1, 2, 3 months, and every 3 months thereafter. The case report forms are uploaded into a web-based database hosted by China Centre for Disease Control (China CDC). Eligibility criteria for this study included: (1) HIV patients that were over 18 years old; (2) had positive HBV surface antigen results at the time of ART initiation; (3) initiated free first-line ART between 2010 and 2014; and (4) provided informed consent. The NFATP treatment criterion since 2010 requires at least one of the following: (1) WHO disease stage 3 or 4 (CD4 count of ≤350 cells/μL); (2) willingness to receive ART irrespective of other criterion, such as high CD4 count when initiating ART^24^. In China, the first-line regimens of AZT/d4T + 3TC + NVP were changed to TDF/AZT + 3TC + EFV/NVP in 2012, as recommended by WHO^24^. This study was approved by the institutional review board of the Guangxi Center for Disease Control and Prevention. All research methods in this study were carried out in accordance with the approved guidelines.

### Data collection

The NFATP observational cohort study included baseline and follow-up characteristics. Baseline characteristics included demographic variables (age, sex, marital status) and clinic variables (route of HIV transmission, HBV infection before ART, HCV infection before ART, CD4 cell count (cells/mm^3^) before ART, WHO clinic stage before ART, initial ART regimen, current ART regimen, year of ART initiation). Follow-up characteristics were evaluated at every 3-months and included death or attrition. Variables collected at each follow-up included transferals to another clinic, cessation of ART, loss to follow-up, duration of ART, and survival status, which were obtained from hospital records or having clinic doctors’ call family members to inquire about death. HBV infection was defined as positive serum samples for Hepatitis B surface antigen (HBsAg) which was tested using an HBV ELISA. Samples were tested for antibodies to HCV by ELISA^6^.

### Statistical analysis

We conducted a prospective follow-up study analysis. Time zero was defined as the date of ART initiation, and data was censored on June 30^th^, 2016. Patients were censored if they were still alive or transferred to another clinic for care. Study endpoints included death and attrition. Attrition included lost to follow-up or withdrawal of ART as recorded in the HIV treatment database. Lost to follow-up was defined as missing more than 90 days after the last date seen in clinic, which was also defined as the date of withdrawal, as previous published studies^9,10^. Mortality and attrition rates were calculated based on Poisson distributions, and reported as deaths and attritions per 100 person-years of follow-up study, respectively.

In order to assess treatment effects of the differential first-line antiretroviral therapy, we classified the initial ART regimens as three groups: the first-line ART containing D4T (d4T + 3TC + NVP), the first-line ART containing AZT (AZT + 3TC + EFV/NVP) and the first-line ART containing TDF (TDF + 3TC + EFV/NVP). We used Cox proportional hazard models to evaluate treatment effects of initial ART regimen on death and attrition (lost to follow-up or withdrawal of ART), respectively. Competing risks for cause-specific hazard models were censored accordingly [9.10]. The adjusted models were used to control for potential bias. The following covariates of the adjusted model were included demographic variables (age, sex, marital status) and clinic variables (route of HIV infection, CD4 count before ART, WHO clinic stage before ART, year-initiated ART). And then, the adjusted model for sub-group analysis of death and attrition was used to compare the ART containing AZT with the ART containing TDF, respectively. A two-sided p-value less than 0.05 was regarded as statistically significant for all analyses. We did the analyses with Statistical Analysis System (SAS 9.1 for Windows; SAS Institute Inc., NC, USA).
